# Etiology of Severe Acute Respiratory Infections, Bangladesh, 2017

**DOI:** 10.3201/eid2701.201347

**Published:** 2021-01

**Authors:** Md R. Rahaman, Karen A. Alroy, Chris A. Van Beneden, Michael S. Friedman, Erin D. Kennedy, Mahmudur Rahman, Arunmozhi Balajee, A.K.M. Muraduzzaman, Tahmina Shirin, Meerjady S. Flora, Eduardo Azziz-Baumgartner

**Affiliations:** The University of Adelaide, Adelaide, South Australia, Australia (M.R. Rahaman);; Institute of Epidemiology, Disease Control, and Research, Dhaka (M.R. Rahaman, A.K.M. Muraduzzaman, T. Shirin, M.S. Flora);; Centers for Disease Control and Prevention, Atlanta, Georgia, USA (K.A. Alroy, C.A. Van Beneden, M.S. Friedman, E.D. Kennedy, A. Balajee, E. Azziz-Baumgartner);; icddr,b, Dhaka, Bangladesh (M.R. Rahman);

**Keywords:** severe, influenza, vaccine, Bangladesh, respiratory infections, vaccine-preventable diseases, viruses

## Abstract

In April 2017, surveillance detected a surge in severe acute respiratory infections (SARI) in Bangladesh. We collected specimens from SARI patients and asymptomatic controls for analysis with multipathogen diagnostic tests. Influenza A(H1N1)pdm09 was associated with the SARI epidemic, suggesting that introducing vaccines and empiric antiviral drugs could be beneficial.

In April 2017, the Institute of Epidemiology Disease Control and Research (IEDCR) in Bangladesh noted an 89% increase in severe acute respiratory infections (SARI) compared with April 2016 through the National Influenza Surveillance Bangladesh (NISB) at 10 tertiary-care hospitals. During April 10–June 21, 2017, we conducted a case–control study to ascertain the cause of the outbreak and its associated risk factors.

We defined a SARI case as acute respiratory illness in a patient within 10 days of onset, with history of fever and cough, and requiring hospitalization (*1*). We sought to enroll all adults >18 years of age who were admitted to NISB hospitals with SARI. Staff screened patients for eligibility, obtained written informed consent, surveyed participants about demographics, and took combined nasal and throat swab samples. Patients who died in hospital wards before enrollment were ineligible. Within 2 days of case-patient enrollment, staff enrolled 2 asymptomatic controls, identified by convenience from the same hospitals’ outpatient clinics, surveyed them, and took combined nasal and throat swab samples. Patients who had fever or respiratory symptoms in the previous 14 days were ineligible to serve as controls. Swab specimens from case-patients and controls were tested for viral, bacterial, and fungal nucleic acids using FTD Respiratory pathogens 33 real-time reverse transcription PCR (Fast Track Diagnostics, http://www.fast-trackdiagnostics.com)(*2*).

We examined the association between SARI case-status and sociodemographic characteristics, preexisting conditions, and pathogens detected through multivariate logistic regressions. The investigation was judged to be public health action by the institutional review boards of the IEDCR and US Centers for Disease Control and Prevention (approval no. IEDCR/IRB/2016/17).

We identified 79 eligible SARI case-patients and 158 eligible controls from 5 NISB hospitals (Figure). Of these, 73 (92%) eligible patients and 146 (92%) eligible controls consented to participate (Table). Case-patients were more likely than controls to be male (89% vs. 77%; p = 0.041 by Fisher exact test) and have preexisting underlying chronic conditions (47% vs. 25%; p = 0.001), including asthma (36% vs. 12%; p = 0.000) and allergies (27% vs. 10%; p = 0.003) (Table). Although 34 (47%) of case-patients were treated with antibiotics, none were treated with antiviral drugs.

**Figure:**
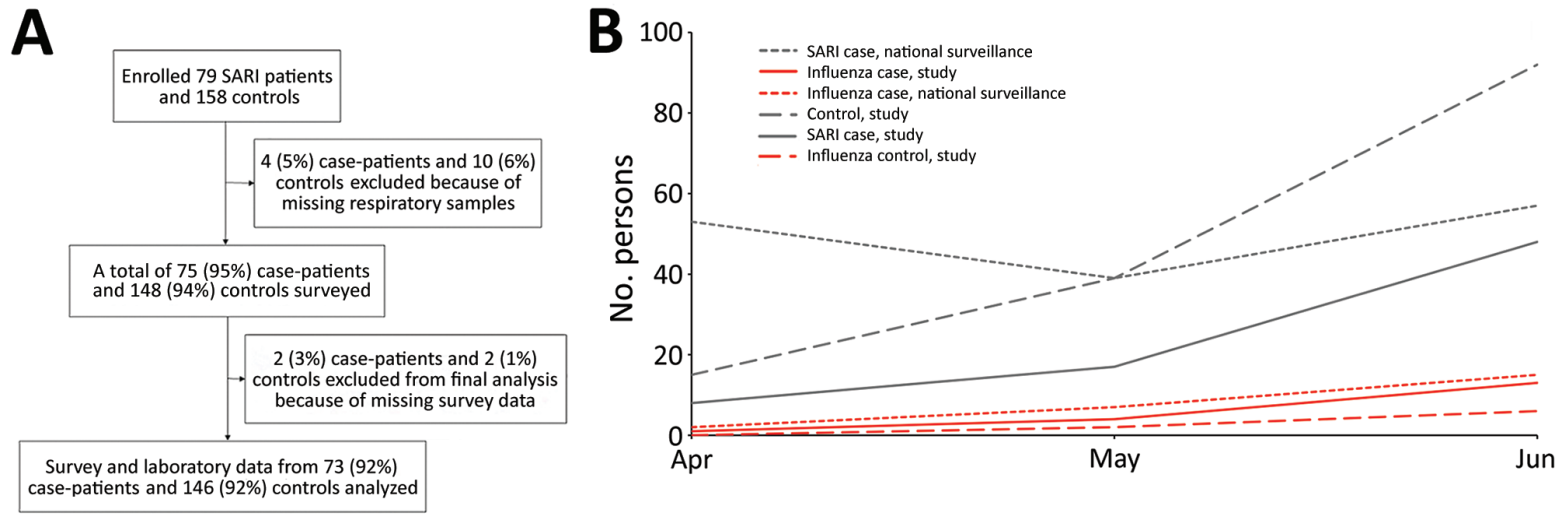
Participant enrollment in study of etiology of severe acute respiratory infections and influenza activities in Bangladesh, April–June, 2017. A) Enrollment of adults with severe acute respiratory infections (SARI) and their corresponding controls. B) Influenza activities, study (April 10−June 21) and national surveillance (April–June). Percentages might not sum to 100 because of rounding.

**Table:** Participant characteristics and their associations with severe acute respiratory infection case–control status*

Characteristics	No. (%)		Unadjusted OR (95% CI)	p value or aOR (95% CI)†
	Case-patients, n = 73	Controls, n = 146		
Age, y, n = 219, median (IQR)	35 (25−50)	35 (24−50)		0.949‡
Sex, n = 219				
F	8 (11)	33 (23)	Referent	
M	65 (89)	113 (77)	2.37 (1.03–5.44)	0.041
Duration from illness onset to seeking care at sentinel hospitals (case-patients), d				
Median (IQR)	4 (3−5)			
Education, n = 218				
Below secondary	50 (68)	103 (71)	Referent	
Secondary and above	23 (32)	42 (29)	1.13 (0.61–2.08)	0.699
Occupation, n = 218				
Unemployed	21 (29)	35 (24)	Referent	
Homemaker	7 (10)	33 (23)	0.35 (0.13–0.94)	0.037
Farmer	13 (18)	9 (6)	2.41 (0.88–6.59)	0.087
Poultry worker	1 (1)	0		
Healthcare worker	0	1 (1)		
Other§	31 (42)	67 (46)	0.77 (0.39–1.54)	0.459
Monthly income, USD, n = 218				
<250¶	68 (93)	126 (86)	Referent	
>250	1 (1)	6 (4)	0.31 (0.04–2.62)	0.281
Refused to answer	4 (5)	13 (9)	0.57 (0.18–1.82)	0.342
No. household members, n = 218				
<7	66 (90)	131 (90)	Referent	
>8	7 (10)	14 (10)	0.99 (0.38–2.58)	0.988
Body mass index# category, n = 176				
Underweight	5 (7)	11 (8)	Referent	
Healthy weight	44 (60)	95 (65)	1.02 (0.33–3.11)	0.974
Overweight	4 (5)	15 (10)	0.59 (0.13–2.70)	0.494
Obese	1 (1)	1 (1)	2.20 (0.11–42.73)	0.602
Behavioral factors				
Current smoker, n = 215	16 (22)	42 (29)	0.69 (0.35–1.33)	0.266
Ever smoker, n = 215	22 (30)	53 (36)	0.77 (0.42–1.41)	0.400
Betel nut chewer, n = 219	12 (16)	26 (18)	0.91 (0.43–1.92)	0.801
Gul user, n = 214**	2 (3)	8 (5)	0.49 (0.10–2.37)	0.374
Self-reported preexisting conditions, n = 219				
Any underlying chronic condition	34 (47)	36 (25)	2.66 (1.47–4.83)	0.001
Asthma	26 (36)	17 (12)	4.17 (2.08–8.36)	0.000
Chronic lung disease††	2 (3)	2 (1)	2.07 (0.29–15.02)	0.471
Diabetes	7 (10)	17 (12)	0.83 (0.33–2.11)	0.695
Neurologic disease	13 (18)	21 (14)	1.26 (0.59–2.69)	0.552
History of allergies, n = 219‡‡	20 (27)	15 (10)	3.11 (1.48–6.56)	0.003
Antibiotics for current illness, case-patients	34 (47)			
Antivirals for current illness, case-patients	0			
Viral pathogens, n = 219				
Influenza viruses	18 (25)	8 (5)	5.65 (2.32–13.74)§§	11.34 (3.74–34.33)§§
Influenza A(H1N1)	12 (16)	3 (2)	9.38 (2.56–34.41)§§	11.38 (2.73–47.42)§§
Unsubtyped influenza A	5 (7)	4 (3)	2.61 (0.68–10.03)	11.22 (1.63–77.23)¶¶
Influenza C	1 (1)	2 (1)	1.00 (0.09–11.21)	1.46 (0.10–20.92)
Parainfluenza viruses	4 (5)	1 (1)	8.41 (0.92–76.62)	
Human rhinovirus	8 (11)	11 (8)	1.51 (0.58–3.94)	1.35 (0.43–4.27)
Human metapneumovirus	6 (8)	5 (3)	2.53 (0.74–8.57)	1.34 (0.34–5.35)
Adenovirus	1 (1)	2 (1)	1.00 (0.09–11.21)	1.84 (0.14–23.36)
Enterovirus	1 (1)	3 (2)	0.66 (0.07–6.48)	0.77 (0.07–8.81)
Human bocavirus	2 (3)	1 (1)	4.08 (0.36–45.80)	2.48 (0.19–32.45)
Coronaviruses	8 (11)	10 (7)	1.67 (0.63–4.44)	1.30 (0.37–4.55)
Bacterial pathogens, n = 219				
* Streptococcus pneumoniae*	9 (12)	32 (22)	0.50 (0.23–1.12)	0.37 (0.14–0.97)¶¶
*Haemophilus influenzae* type b	0	4 (3)		
* Staphylococcus aureus*	10 (14)	16 (11)	1.29 (0.55–3.00)	1.96 (0.75–5.16)
* Moraxella catarrhalis*	3 (4)	5 (3)	1.21 (0.28–5.20)	0.38 (0.06–2.53)
*Bordetella* spp.	2 (3)	3 (2)	1.34 (0.22–8.22)	1.73 (0.19–15.92)
* Klebsiella pneumoniae*	23 (32)	34 (23)	1.52 (0.81–2.83)	1.21 (0.59–2.47)
*H. influenzae* other than type b	9 (12)	24 (16)	0.71 (0.31–1.63)	0.69 (0.27–1.77)
* Mycoplasma pneumoniae*	0	1 (1)		
No pathogens detected	20 (27)	54 (37)	0.64 (0.35–1.19)	0.70 (0.35–1.39)

*Percentages, where relevant, might not sum to 100 because of rounding. aOR, adjusted odds ratio; IQR, interquartile range; OR, odds ratio. †Adjusted for age, sex, and past medical history, including history of asthma and allergies. ‡Calculated by Wilcoxon rank-sum test. §Other includes day laborer, butcher, businessman, office worker, and any other occupation not listed. ¶Per capita income in Bangladesh in 2015 was $1,314 (*3*). #Healthy body mass Index range is 18.5–24.9 (*4*). **Smokeless tobacco constituted of tobacco powder and molasses (*5*). ††Includes all self-reported lung diseases other than asthma of >3 months’ duration. ‡‡History of allergies included food allergy (n = 21), dust allergy (n = 6), drug allergy (n = 1), and nonspecified allergy (n = 7). §§p<0.001. ¶¶p<0.05.

Fifty-three (73%) case-patients and 92 (63%) controls tested positive for >1 pathogens (Table). Among 53 test-positive case-patients, 18 (25%) tested positive for influenza viruses (Figure), including 12 (67%) for influenza A(H1N1)pdm09, 5 (28%) for unsubtyped influenza A, and 1 (6%) for influenza C. Among 92 test-positive controls, 8 (5%) tested positive for influenza viruses (Figure), including 3 (38%) for influenza A(H1N1), 4 (50%) for unsubtyped influenza A, and 2 (25%) for influenza C, 1 of whom had a codetection of an unsubtyped influenza A virus.

Male sex (odds ratio [OR] 2.4, 95% CI 1.0−5.4), >1 preexisting conditions (OR 2.7, 95% CI 1.5−4.8), asthma (OR 4.2, 95% CI 2.1−8.4), and history of allergies (OR 3.1, 95% CI 1.5−6.6) were more common among SARI case-patients than controls (Table). Any influenza virus (OR 5.7, 95% CI 2.3−13.7), and influenza A(H1N1) specifically (OR 9.4, 95% CI 2.6−34.4), was significantly associated with SARI status. Only influenza A(H1N1) (OR 11.4, 95% CI 2.7−47.4) remained associated with case status.

The surge in SARI during April 2017 was attributable to influenza viruses. Although influenza epidemics in Bangladesh typically occur during May–September (*6*), our investigation suggests the 2017 season started a month early. The government of Bangladesh has purchased Northern Hemisphere formulation influenza vaccines for Hajjis (i.e., for persons entering Mecca) (*7*,*8*) but does not otherwise have a vaccination policy because its possible benefit has not been shown in Bangladesh. Influenza vaccines are sporadically available in Bangladesh’s private market but rarely used. Our findings suggest the potential utility of estimating the cost-benefit ratio of influenza vaccination among persons at high risk for SARI using Southern Hemisphere formulations, which incorporate the most current vaccine strains recommended by the World Health Organization (*7*) before the April–May start of the Bangladesh influenza season.

Patients with SARI were frequently prescribed antibiotics but not antiviral drugs. Whether antibiotics benefit SARI patients or solely drive antibiotic resistance is unclear; however, observational data suggest the benefit of empiric antiviral drugs to prevent influenza complications during epidemics (*9*). IEDCR recommended empiric treatment of patients with SARI with antiviral drugs during the 2009 pandemic but does not currently (*10*). Quantifiying the cost-effectiveness of empiric antiviral treatment for SARI patients during influenza epidemics could be valuable, particularly when used within 48 hours of symptom onset, when antivirals are most effective (*6*,*9*).

 This investigation had limitations. Controls were not randomly selected, and age and sex were not matched with case-patients. We limited our sample collection to the upper respiratory tract, did not have wells to identify influenza A(H3N2) RNA, and did not collect blood or other samples that would have more accurately detected bacterial infections.

Our investigation of a surge in SARI cases during April 2017 in Bangladesh suggests the value of surveillance and rapid response in identifying the etiology of outbreaks. Our findings also suggest the potential value of estimating the cost-effectiveness of influenza vaccination campaigns among groups at high risk for SARI administered as early as late March to early April and of empiric antivirals during Bangladesh’s influenza epidemics.
